# The Puzzle of Coccoid Forms of *Helicobacter pylori*: Beyond Basic Science

**DOI:** 10.3390/antibiotics9060293

**Published:** 2020-05-31

**Authors:** Enzo Ierardi, Giuseppe Losurdo, Alessia Mileti, Rosa Paolillo, Floriana Giorgio, Mariabeatrice Principi, Alfredo Di Leo

**Affiliations:** 1 Section of Gastroenterology, Department of Emergency and Organ Transplantation, University “Aldo Moro” of Bari, 70124 Bari, Italy; giuseppelos@alice.it (G.L.); alessia.mileti@hotmail.it (A.M.); rosina.paolillo@gmail.com (R.P.); b.principi@gmail.com (M.P.); alfredo.dileo@uniba.it (A.D.L.); 2 Ph.D. Course in Organs and Tissues Transplantation and Cellular Therapies, Department of Emergency and Organ Transplantation, University “Aldo Moro” of Bari, 70124 Bari, Italy; 3 THD S.p.A., 42015 Correggio (RE), Italy; flomic@libero.it

**Keywords:** *Helicobacter pylori*, coccoid form, viable non culturable, antibiotic resistance, real time polymerase chain reaction, therapy

## Abstract

*Helicobacter pylori* (*H. pylori*) may enter a non-replicative, non-culturable, low metabolically active state, the so-called coccoid form, to survive in extreme environmental conditions. Since coccoid forms are not susceptible to antibiotics, they could represent a cause of therapy failure even in the absence of antibiotic resistance, i.e., relapse within one year. Furthermore, coccoid forms may colonize and infect the gastric mucosa in animal models and induce specific antibodies in animals and humans. Their detection is hard, since they are not culturable. Techniques, such as electron microscopy, polymerase chain reaction, loop-mediated isothermal amplification, flow cytometry and metagenomics, are promising even if current evidence is limited. Among the options for the treatment, some strategies have been suggested, such as a very high proton pump inhibitor dose, high-dose dual therapy, *N*-acetycysteine, linolenic acid and vonoprazan. These clinical, diagnostic and therapeutic uncertainties will represent fascinating challenges in the future.

## 1. Introduction

*Helicobacter pylori* (*H. pylori*) is a Gram-negative bacterium able to colonize the human stomach, where it is the most common cause of chronic active gastritis, peptic ulcer [1], adenocarcinoma [2], mucosal associated lymphoid tissue (MALT) lymphoma [3] and extra-gastric diseases [4]. In all these conditions, bacterium eradication plays a key role for disorder treatment or prevention.

The spreading of antibiotic resistance is the main reason for the progressive increase of therapy failures, which at the moment represent a hot topic in many countries [5]. Indeed, in this regard a lot of studies have been focused on novel antibiotic regimens [6] and suitable tests for resistance detection, especially based on molecular methods in the light of technical difficulties of culture-based approaches [7]. In addition, culture-based resistance evaluation requires a long period to obtain a result and, therefore, their use is recommended only after at least two therapeutic regimen failures.

Nevertheless, there are circumstances where a treatment failure might not be secondary to antibiotic resistances [8]. In these cases, the survival of the organism in the coccoid form has been regarded as a possible explanation.

On these bases, we carried out a narrative review in order to deal with the poorly explored setting of the biology and clinical impact of coccoid forms in the wide scenario of *H. pylori* infection.

## 2. Coccoid Forms of *H. pylori*

Similar to some other microorganisms, *H. pylori* is able to modify its morphology to survive in many adverse environmental conditions such as antibiotics, temperature, pH and increased oxygen tension [9,10].

**Figure 1:**
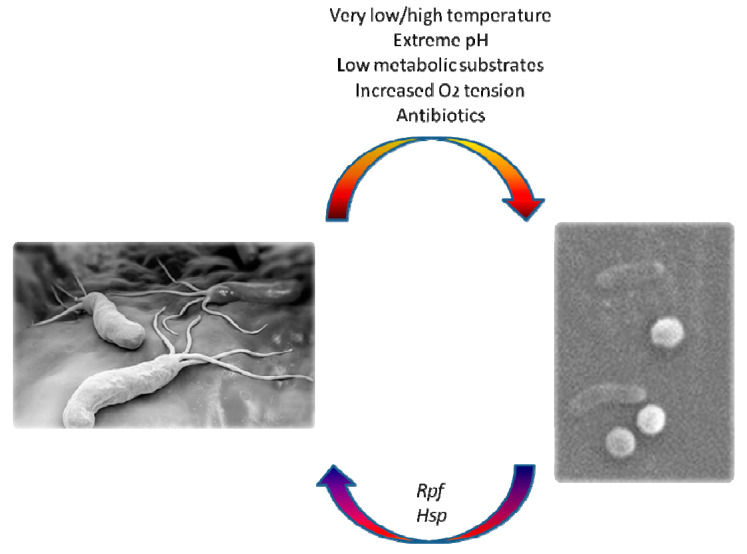
Transition from spiral-shaped viable form of *H. pylori* to viable but non culturable (VBNC) coccoid forms (scanning electron microscope) and factors affecting this process.

It can enter a “viable but non culturable” (VBNC) state, that can be defined as an inactive form of life induced by stressful conditions. This state is characterized by a metabolic blackout and a diversion from the classical bacterial spiral shape, which is crucial for gastric colonization and cork-screwing penetration of dense mucus covering the wall of this organ. Thus, the microbe takes on a coccoid appearance (Figure 1) while retaining its active virulence factors with minimal metabolic activity; VBNC organisms express their genes at low levels and cannot be grown using conventional culture techniques [9,10,11].

Bacteria can remain in VBNC state for over one year, but when environmental conditions become favorable, they can acquire again their spiral shape, thus becoming metabolically active and culturable [9,10,11].

## 3. Biology and Immunology

Even if coccoid forms have been considered in the past as the result of degenerative events, nowadays it is known that this transformation is an active mechanism of protection by an adaptation process [11,12]. The data available from the literature describe the existence of three metabolic forms of *H. pylori* (from the most to the least virulent)as follows: viable culturable bacillary spiral, VBNC coccoid and non-viable degenerative forms [13,14]. In the same way, three morphological types of *H. pylori* have been described: S-shaped (spiral), coccoid and U/C-shaped forms, which are intermediate/transitional organisms. During the conversion from bacillary spiral to coccoid appearance, scanning electron microscopy (SEM) shows that the bacterium proceeds through intermediate shapes. In this phase, flagella enwrap the coccoid cellular structure and become invisible [15]. At first, the protoplasmic matrix shrinks and periplasm increases at the opposite side from the flagella basal complex. Furthermore, a stretching of the cell wall occurs with the accumulation of matrix to the periphery leading to U/C-shaped cell [16]. The final change into coccoid forms provides for the possibility of two sub-types: subtype A with irregular side and rugged surface (the dead cell) and subtype B with smooth surface (the living cell).

Bacterial inactive phases, identified by the term of “dormancy”, represent a reversible state, in which the cells display a low metabolic activity and do not replicate for a long period. This state enhances cell resistance and favors their survival [17,18,19]. The ability of *H. pylori* to enter a dormant state has epidemiological implications, because it can be considered potentially capable of bacterial re-infections and spreading [20]. Indeed, despite this hibernating condition, bacteria retain a low urease activity and expression of urease encoding genes are detectable by polymerase chain reaction (PCR) [21,22,23].

Intermediate and coccoid forms can occur in culture or gastric mucosa depending on exposure to unfavorable factors [24,25,26,27,28]. Mouery et al. [29] studied the graphical distribution ratio of these forms in culture after 12, 24 and 48 h and showed that the number of coccoid forms increased with the time. In human gastric mucosa, *H. pylori* becomes coccoid under anti-secretory or anti-bacterial drugs exposition. Khomeriki et al. [30] found that spiral bacteria take a few hours to transform itself into coccoid form, after adhesion to the surface of gastric epithelium. On the other hand, in the absence of antibiotics or anti-secretory drugs, *H. pylori* can enter a transitional or coccoid form due to the accumulation of toxic metabolic products, such as reactive oxygen species, or the presence of specific pyrimidine nucleotides.

It is known that bacterial cytokines can stimulate the “resuscitation” of a dormant bacterial form. In this regard, Mukamolova et al. [31] isolated a 16–17 kDa protein (a pheromone derived from bacterial cytokines), named resuscitation-promoting factor (Rpf) in cultivation of *Micrococcus luteus*. Nevertheless, it is not yet known whether there are cytokines able to reactivate *H. pylori*. Heat shock protein (Hsp) has been hypothesized to be a trigger for reactivation of the dormant bacteria for its trophic effect and ability to induce modifications of cell cytoskeleton. Accordingly, it is possible that a condition of mild acidosis (pH 3.5–5), may induce protein synthesis in coccoid and spiral forms [25,26,27,28,29,30,31,32]. These proteins are part of the chaperones family, proteins that support the folding or unfolding of supplementary high-molecular-weight proteins. The most studied *H. pylori* chaperones are HspA and HspB. They are essential for urease synthesis and induce lymphocyte activation, cytokine and chemokine expression and apoptosis. Moreover, they might also stimulate an autoimmune response because of their high antigenic similarity with some structures of gastric mucosa [33].

Currently, little is known about the pathogenicity of coccoid forms and whether these forms may provoke gastrointestinal diseases. Some studies reported that the percentage of coccoid form is higher in the duodenum than in the stomach [34,35]. In fact, in vitro and in vivo experiments proved that coccoid forms may survive in alkaline pH as well as in aerobiosis, high temperatures, prolonged incubation in water and under treatments with proton pump inhibitors or antibiotics [36,37,38,39] by maintaining a minimum detectable level of urease activity [40] and producing very small amounts of proteins and DNA [25,36]. On the other hand, coccoid forms, as above reported, express all genes of major virulence genes (ureA, ureB, hpaA, vacA, cagA, cagE, BabA) [41]. SEM studies showed that coccoid forms are able to invade gastric epithelium through cellular adhesion and enclosure in vesicles by double layer membrane with consequent cellular enlargement and vulnerability [42,43]. In addition, coccoid bacteria may spread, thus infecting nearby cells and inducing erosions of the mucosal layer [44].

Apart from pathogenic factors, a differential expression of surface antigens between coccoid and spiral forms may exist. Nevertheless, this topic does not show a univocal result in literature. Some authors found no difference in analysis of surface protein between coccoid and spiral forms by two dimensional electrophoresis and immunoblot analysis [45]. On the other hand, other investigations identified a change of 16 out of 30 surface proteins during the conversion into the coccoid form, with a reduction of porin and adhesin [46]. Finally, a protein pattern difference appeared at 97.4 to 45 and 30 kDa molecular weight bands in another study [10].

It is known that *H. pylori* infection causes a local immune reaction that leads to chronic gastric diseases. In order to detect immunogenicity of coccoid forms, Figueroa et al. [47] used a specific enzyme-linked immunosorbent assay (ELISA) technique to evaluate their interaction with a panel of sera from infected individuals: all tested serum panels were reactive with both coccoid and bacillary *H. pylori* preparations. A similar study was performed in children with epigastric pain, where the seroprevalence of antigens prepared from spiral and coccoid morphological forms was examined by ELISA.A fourfold increase in seropositivity for coccoid compared to spiral-form antigens was observed [48]. In this study, antigens of spiral and coccoid forms were prepared by acid glycine extraction from 3- and 150-day-old cultures. The sera were diluted (1:100) and allowed to react with the antigen and revealed by ELISA. Therefore, antigens and antibodies were in-home realized and are not reproducible. Another study by Cellini et al. [49] proved that bacteria were detectable in gastric mucosa two weeks after inoculating suspensions of the coccoid form of *H. pylori* into the stomach of BALB/c mice and histo-pathological changes occurred one month later. Furthermore, all colonized mice exhibited a systemic antibody response to *H. pylori*. Similar experiments showed pathological changes in the stomach of colonized mice and compared the virulence between spiral and coccoid forms of *H. pylori*. She et al. [50] treateda group of BALB/c mice with the bacillary and another with coccoid form and less severe mucosal lesions were seen in mice infected by coccoid organisms. A similar result is reported by Rabelo-Goncalves et al. [51]. Interestingly, some studies demonstrated that the coccoid forms in drinking water provoked a chronic inflammatory process characterized by the development of lymphocytic plaques in Wistar mice [52].

In conclusion, there is evidence that coccoid forms of *H. pylori* may colonize and infect the gastric mucosa in animal models and induce the development of specific antibodies in animals and humans. These phenomena are presumably linked to the ability to retain a low urease activity as well as urease and major virulence factor-encoding genes even in state of “dormancy” characterized by a low metabolic activity and lack of replication.

## 4. Diagnostic Potential

*H. pylori* coccoid forms are viable but non-culturable, therefore it is quite hard to detect them by conventional methods [53]. Some researchers attempted to create a culture-specific medium to grow coccoid forms by exposing the germ to an acid shock in 100-day culture [54] or adding bismuth, amoxicillin and erythromycin to the medium [55] or changing pH [56]. However, results were disappointing, therefore alternative methods have been developed.

Autoradiography is a technique by which an image is produced from the distribution of a radioactive substance as a consequence of decay emissions (e.g., beta particles or gamma rays). This methodology has been applied to reveal *H. pylori* coccoid forms. For this purpose, four strains of *H. pylori* were used to obtain a microenvironment containing water suspensions of 72-h cultured colonies. They were incubated with (3H)-thymidine for 24–72 h. Autoradiography of tritium-labeled cells of *H. pylori* showed an accumulation of silver grains in coccoid forms due to the uptake of radio-labeled substrates into bacterial DNA. Additionally, it was demonstrated that bacteria could remain viable at 4 °C for 26 months [57].

Electron microscopy may directly visualize coccoid forms. In this regard, Willen et al. [58] demonstrated that both scanning and transmission electron microscopy are able to show the transition from spiral to coccoid form and vice versa.

Fluorescent in situ hybridization relies on ribosomal RNA oligonucleotidic probes, thus realizing a fast and sensitive tool, which has been used for coccoid form detection in water samples, where probes were targeted toward 16S rRNA and ureA/B genes [59], and raw bovine milk [60].

Nevertheless, techniques based on molecular biology, able to detect and amplify bacterial genome, are considered as the gold standard nowadays for their feasibility and reliability. PCR and real time (RT)-PCR are the most widespread ones, because they have the advantage to distinguish the organisms even in low numbers and in a non-replicative phase [61]. Usually, some target genes such as ureC, ureA or glmM have been employed; ureC seems to be the most promising one [62,63,64]. Nevertheless, many different probes have been used. Janzon et al. [65] demonstrated the presence of coccoid forms in water by amplifying hpaA and glmM genes. Sen et al. [66] elaborated an internal control for evaluation and standardization of a PCR assay for *H. pylori* analysis in drinking water using 135-bp modified at the probe binding region amplicon and incorporated into a single-copy plasmid of *E. coli*. Lastly, a 6-carboxyfluorescein-labeled probe was selected to improve *H. pylori* amplification in some unfavorable media [67]. In conclusion, *H. pylori* coccoid form PCR assay has several pros, since it does not rely on culturing, whose feasibility is doubtful. Furthermore, several samples can be analyzed quickly and together. Finally, real-time PCR can bypass many human causes of error due to possible contamination or processing mistakes. The only drawback of PCR is that it cannot distinguish between living or dead organisms.

Probably, the most interesting novelty is loop-mediated isothermal amplification (LAMP). It amplifies targeted DNA producing magnesium pyrophosphate. This molecule is then assessed by photometry, since the pyrophosphate increases the turbidity of the solution [68]. This technique has been applied to *H. pylori* [69] and showed a very high accuracy, speed, and sensitivity both in water and stomach biopsy samples [70]. It has been applied to glmM and vacA genes [68] with promising results since it is fast and accurate in detecting bacterial DNA, even though its usefulness to distinguish coccoid forms will be addressed only in the future.

In comparison with PCR, flow cytometry is not influenced by the drawback of inability to distinguish viable from dead cells. Indeed, it can discriminate among reproductively viable, metabolically active, intact and permeabilized *H. pylori*. Therefore, it could be able to estimate the proportion of coccoid germs out of the whole *H. pylori* charge [71]. So, cytometry might provide some further information about qualitative bacterial state, although a rapid and standardized approach is not available at the moment.

Finally, the most recent frontier is represented by metagenomic approaches. This is very important for coccoid forms since these techniques are able to study nonculturable microorganisms [72]. To the best of our knowledge, few studies have applied metagenomics to *H. pylori*. Zheng et al. [73] showed a method by which metagenomic analysis of *H. pylori* was possible in paraffin embedded biopsy samples. Moreno-Mesonero et al. [74] attempted to detect the germ in water samples by this technique. However, despite being promising, the reports are scant and the scope of metagenomics has still to be explored.

In conclusion, at this time we do not have a simple diagnostic test for the coccoid forms of *H. pylori* that may be reproducible on a large scale and feasible in daily practice. Electron microscopy and PCR seem to be the most suitable methods. The first, however, is not widely available, whilst the second does not offer a conclusive agreement about standardization and reproducibility of the technique. Additionally, electron scanning microscopy still remains time consuming, therefore molecular biology techniques are becoming the most attractive tools. Nevertheless, in this particular case, due to the absence of a gene that is specifically expressed in dormant forms, the positivity of PCR along with negativity of traditional methods that detect metabolically active bacterium (such as urea breath and rapid urease tests), could be the key solution

## 5. Clinical Relevance

As mentioned above, coccoid forms, although less virulent than spiral ones and with a low metabolic activity, are able to colonize and induce inflammation of gastric mucosa since they express urease, cytotoxic islands and vacuolating toxin genes [75,76]. Additionally, they are also poorly responsive to antibiotic therapy [77]. Therefore, the administration of antisecretory and antibacterial drugs can lead to the conversion of spiral into coccoid forms of *H. pylori*. They might be able to facilitate a reverse transition into the replicative state and be involved in the recurrence of peptic ulcer disease. Indeed, the simultaneous presence of chronic gastritis and the same strain of *H. pylori* one year after therapy in patients with peptic ulcer may suggest that the bacterium has undergone a transformation from a dormant state into the replicative form. Consequently, for a successful therapy, it could be essential to eradicate not only spiral, but also viable dormant bacteria.

As reported, the induction of reversion may occur under the influence of specific molecules, such as HspA, HspB or Hsp [78]. On the other hand, similarly to bacillary spiral forms, coccoid ones induce a humoral immune response that is associated to chronic gastric disease. This process, in the presence of specific molecules inducing coccoid reversion to spiral forms, may induce *H. pylori* related autoimmune phenomena especially at the level of parietal cell canalicula with a consequent progression of bacterial to autoimmune gastritis [79,80].

Based on the above, two types of recurrence of *H. pylori* infection after a successful eradication may be recognized:

- “Relapse”: the bacterium responsible for the recurrence is genetically the same as that identified before the eradication and, relapse usually occurs within the first year following the eradication;
- “Re-infection”: the bacterium causing the recurrence is different from that identified before the eradication and develops a long time after the first eradication.

Therefore, the analysis of genetic polymorphisms by PCR is necessary to differentiate relapse from re-infection.

Relapse is mainly due to the same bacterium of the first infection and might occur by the transformation of spiral forms of *H. pylori* into coccoid ones resistant to antibiotics with a successive reversion as well as by the development of a biofilm inaccessible to antibiotics that is able to surround and protect coccoid bacteria [81,82,83].

Unfortunately, most of studies on coccoid forms are basic science articles, therefore there is no sufficient data to understand in which cases it would be useful to look for these forms.

## 6. Possible Estimation of Problem Dimension: Preliminary Experience

Recently, RT-PCR has been widely used as a relevant diagnostic tool for several bacterial and viral infections for its ability to amplify small amounts of specific genetic sequences with high sensitivity. Its application in the diagnosis of *H. pylori* infection has been well established and the amplification of the 23S rRNA sequence is the most used method for both bacterial DNA detection and mutations characterizing resistance to clarithromycin. Mutations conferring clarithromycin resistance have a rate of about 20%–30%. This value is in agreement with the data coming from our geographic area, and several mutations (mainly A2143G, A2142G and A2142C) may occur, with possibility of heteroresistance and multiple resistance [84,85]. In the previous experience of our group, *H. pylori* DNA sequences were found in three out of fifty histology-negative cases, thus suggesting that RT-PCR may be a key tool for refining bacterial detection by both clarifying the diagnosis in doubtful cases and guiding a successful therapeutic regimen by detecting antibiotic resistances [86]. After this preliminary experience, a noninvasive molecular test with high sensitivity and specificity (THD fecal test) was developed to detect DNA-specific sequences and point mutations in the stools of patients [85,87].

It is possible that the presence of *H. pylori* DNA in the absence of positive conventional tests (histology, rapid urease, stool antigen and urea breath test) may reflect the presence of coccoid forms. In Table 1, we report unpublished data obtained in a series of 185 *H. pylori* positive dyspeptic subjects, in whom at least one therapeutic antibiotic regimen had failed. Six patients (3.3%) showed a positive molecular test with negative conventional tests. Two of them, moreover, showed point mutations characterizing clarithromycin resistance. Overall prevalence of clarithromycin resistance was 24.3% in the whole series, thus explaining about one fourth of failures.

These preliminary data, even if speculative, suggest that the role of coccoid forms in antibiotic *H. pylori* therapy failure could be small when compared to antibiotic resistances.

## 7. Therapeutic Prospective Options

The therapy of *H. pylori* infection in the bacillary form is based on the recommendations of many guidelines, such as European and Italian ones [4,88]. In detail, in areas with high resistance to clarithromycin (>15%), quadruple therapy with or without (concomitant/sequential) bismuth are suggested. These regimens have been empirically formulated taking into account the disappointing decreasing success rate of previously widely used schemes, especially triple therapies. The checks for therapy outcomes require a non-invasive test such as a urea breath test at least 4–8 weeks after the end of treatment.

A study by Figura et al. [83] showed that some factors can affect the response to therapy. This study showed that patients with peptic ulcers have a higher eradication rate than those with dyspepsia. A possible explanation is that the ulceration increases the spread of antibiotics allowing their penetration as well as the high density of inflammatory cells induces vascular alterations that increase epithelial permeability. However, the main result of this study was that, in patients who did not reach eradication, the presence of coccoid forms of *H. pylori* in the biofilm could be invoked without antibiotic resistance. In addition, the study found that successful eradication correlated with *H. pylori* CagA-positive strains; this may occur for the following reasons: i. CagA-positive *H. pylori* may induce peptic ulcer more frequently; ii. bacteria expressing *cagA* show a fast growth and, since many antibiotics interfere with cell division, these bacteria are more prone to be destroyed; iii. bacteria that express CagA induce the production of pro-inflammatory cytokines, such as interleukin 1 β or tumor necrosis factor α, which are powerful inhibitors of acid secretion and therefore increase the effectiveness of the treatment [89]. Similar results were reported by Russo et al. [90] in a study from Southern Italy.

The therapeutic strategies that have been proposed to overcome the ineffectiveness of antibiotics on coccoid forms are summarized as follows:

- Increase of acid inhibition: this option may be carried out in three different ways:
  - High-dose dual therapy consisting of the combination of amoxicillin (e.g, 1 g tid or 750 mg qid) and a proton pump inhibitor (standard-dose tid or qid or double standard-dose bid) for 14 days. This approach is based on previous studies showing that this regimen has achieved a satisfactory therapeutic outcome as empirical first-line or rescue therapy for *H. pylori* infection [91].
  - Increase of proton pump inhibitor (PPI) dose. This assumption fits with the fact that the reproduction cycle of *H. pylori* is dependent on gastric pH, since it enters the replicative cycle at neutral pH below the gastric mucus layer and turns into coccoid forms at acidic pH. In this setting, not only the dose, but also the choice of PPI might have relevance. In fact, it is presumable that second-generation PPIs (esomeprazole, rabeprazole) are more effective than first-generation PPIs (omeprazole, lansoprazole). The explanation may be that second-generation drugs have a metabolism less dependent on CYP2C19 allelic variants and, therefore, active metabolite serum levels are more stable over time [92].
  - Use of Vonoprazan (VPZ). VPZ is a new, powerful acid-inhibitory drug. It is a competitive inhibitor of H+/K+ ATPase pump located on the apical membrane of gastric parietal cells. VPZ became clinically available in Japan in 2015. Compared to conventional PPIs, gastric acid was inhibited by VPZ more rapidly, more strongly and for a longer duration. Moreover, VPZ does not require pharmacological activation and has a protracted half-life. A study reported that VPZ (20 mg bid) potently suppressed acid for 24 h [93]. The Tokyo Helicobacter pylori Study Group showed that the *H. pylori* eradication rate in the third-line treatment was higher in VPZ-based therapy than in PPI-based therapy [93,94]. Moreover, it has been shown that VPZ could increase the eradication rate of triple therapy even in clarithromycin-resistant strains [94]; therefore, it is presumable that VPZ could help the transition of *H. pylori* from coccoid to spiral shape. However, VPZ is currently available only on the Japanese market, and other confirmatory studies are necessary to assess its potential in Western countries [95].
- Free fatty acids. A very recent study has shown that free fatty acids, such as linolenic acid and liposomal linolenic acid, have a bactericidal effect “in vitro” on both bacillary and coccoid forms, regardless of the resistance to antibiotics [96]. Linolenic acid may be effective because it enhances membrane permeability, therefore facilitates cell lysis and antibiotic access into the cells [97].
- Mucolytic substances. Similar to what happens for other bacteria, such as *Pseudomonas aeruginosa*, *Staphylococcus epidermidis* and *E. coli*, the production of biofilm by *H. pylori* is known to facilitate antibiotic resistance. Biofilm is a complex composed by bacteria and extracellular matrix of polyanionic polysaccharides. *H. pylori* coccoid forms may be hidden within biofilm. Some studies demonstrated, in this context, the beneficial role of N-acetylcysteine for its mucolytic and also bacteriostatic properties. On these bases, N-acetylcysteine could be used as a pre-treatment followed by antibiotic therapy [98,99,100].

In conclusion, a proven effective treatment of coccoid forms of H. pylori does not exist. Taking into account their potential role in the recurrence of infection, novel therapeutic regimens should be aimed not only to eradicate the spiral, but also coccoid forms of *H. pylori*. In this context, strategies to treat coccoid forms are only suggestions, since there are no randomized clinical trials. Consequently, we could speculate that free fatty acids or N-acetylcysteine could work as add-on treatments during traditional antibiotic therapy to increase the probability of eradication; however, prospective interventional trials are noticeably necessary.

## 8. Concluding Remarks

*H. pylori* is able to modify its morphology to survive in many adverse environmental conditions by entering a “viable but non culturable” state. The microbe takes on a coccoid appearance even if it retains its active virulence factors with minimal metabolic activity and expresses virulence genes at low levels. When environmental conditions become favorable, they can acquire their spiral shape again, thus becoming metabolically active and develop infection related disorders. This event is defined with the term “Relapse”. So, the conversion of spiral into coccoid forms becomes a factor of treatment failure other than antibiotic resistances. Furthermore, there are evidences that coccoid forms of *H. pylori* may colonize and infect the gastric mucosa in animal models as well as induce the development of specific antibodies in animals and humans. On these bases it is presumable that the clinical relevance of coccoid forms may have been underestimated so far. An additional problem may be related to the evidence that, at this time, we do not have a simple reproducible and feasible diagnostic test to detect coccoid forms. Electron microscopy and PCR seem to be the most suitable methods. The first, however, is not widely available, whilst the second does not offer a conclusive agreement about standardization and reproducibility of the technique. In our experience, the diffusion of noninvasive molecular tests with high sensitivity and specificity able to detect DNA-specific sequences and its mutations in the stools of patients may be promising in this setting. Indeed, in our series, subjects with negative conventional tests and positive DNA may reflect the presence of coccoid forms. Finally, a negative aspect is represented by the lack of specific treatment to eradicate the coccoid forms from human stomach. A very strong acid inhibition and a mucolytic preventive treatment could represent the most promising approaches to overcome the ineffectiveness of antibiotics on coccoid forms.

We believe that the context in which coccoid form assessment could be attractive is multiple failure patients: in these subjects such forms could be more common and their detection could allow the personalization of therapy and enable patients to regain an antimicrobial susceptibility that was only apparent in previous eradication attempts. Additionally, strategies to treat coccoid forms are only suggestions, since there have been no randomized clinical trials. However, we believe that this field is worthy of investigation because it will lead to personalized treatment and optimize the eradication of the bacterium in hard-to-treat cases.

In conclusion, the problem of *H. pylori* false eradications linked to its switch into coccoid forms needs: i. clinical parameters to establish when this “transformation” must be detected, ii. laboratory feasible and reproducible laboratory tests to establish how the detection must be performed, iii. therapeutic strategies able to avoid the survival of the bacterium after conventional treatments. These aspects will represent a fascinating challenge in the near future.

## Figures and Tables

**Table 1:** Data from a series of 185 *H. pylori* positive dyspeptic subjects, in whom at least one therapeutic antibiotic regimen had failed. The combination of negative conventional tests/positive DNA may reflect the presence of coccoid forms.

	No.	%
Total patients	185	100.0
Positivity of at least two conventional tests and DNA	179	96.7
Negative conventional tests with positive DNA (coccoid?)	6	3.3
Overall clarithromycin resistance	45	24.3
Clarithromycin resistance in negative conventional tests/positive DNA	2	1.1
